# Adherence to 5-aminosalicylic acid maintenance treatment in young people with ulcerative colitis: a retrospective cohort study in primary care

**DOI:** 10.3399/BJGP.2023.0006

**Published:** 2023-09-05

**Authors:** Nishani Jayasooriya, Richard C Pollok, Jonathan Blackwell, Alex Bottle, Irene Petersen, Hanna Creese, Sonia Saxena

**Affiliations:** Department of Gastroenterology, St George’s University Hospitals NHS Foundation Trust, London, UK.; Department of Gastroenterology, St George’s University Hospitals NHS Foundation Trust, London, UK.; Western General Hospital, Edinburgh, UK.; School of Public Health, Imperial College London, London, UK.; Department of Primary Care and Population Health, University College London, London, UK; Department of Clinical Epidemiology, Aarhus University, Aarhus, Denmark.; School of Public Health, Imperial College London, London, UK.; School of Public Health, Imperial College London, London, UK.

**Keywords:** 5-aminosalicylic acid, adherence, adolescent, discontinuation, ulcerative colitis, young adult

## Abstract

**Background:**

Maintenance treatment with 5-aminosalicylic acid (5-ASA) is recommended in ulcerative colitis (UC), but accurate estimates of discontinuation and adherence in adolescents transitioning to young adulthood are lacking.

**Aim:**

To determine rates and risk factors for discontinuation and adherence to oral 5-ASA in adolescents and young adults 1 year following diagnosis of UC.

**Design and setting:**

Observational cohort study using the UK Clinical Practice Research Datalink among adolescents and young adults (aged 10–24 years) diagnosed with UC between 1 January 1998 and 1 May 2016.

**Method:**

Time to oral 5-ASA discontinuation (days) and adherence rates (proportion of days covered) were calculated during the first year of treatment using Kaplan–Meier survival analysis. Cox regression models were built to estimate the impact of sociodemographic and health-related risk factors.

**Results:**

Among 607 adolescents and young adults starting oral 5-ASA maintenance treatment, one-quarter (*n* = 152) discontinued within 1 month and two- thirds (*n* = 419) within 1 year. Discontinuation was higher among those aged 18–24 years (74%) than younger age groups (61% and 56% in those aged 10–14 and 15–17 years, respectively). Adherence was lower among young adults than adolescents (69% in those aged 18–24 years versus 80% in those aged 10–14 years). Residents in deprived versus affluent postcodes were more likely to discontinue treatment (adjusted hazard ratio [aHR] 1.46, 95% confidence interval [CI] = 1.10 to 1.92). Early corticosteroid use for an acute flare lowered the likelihood of oral 5-ASA discontinuation (aHR 0.68, 95% CI = 0.51 to 0.90).

**Conclusion:**

The first year of starting long-term therapies in adolescents and young adults diagnosed with UC is a critical window for active follow-up of maintenance treatment, particularly in those aged 18–24 years and those living in deprived postcodes.

## INTRODUCTION

The lifelong condition ulcerative colitis (UC) requires maintenance treatment in primary care; however, compliance can often be suboptimal in younger populations than in adults.^1^ Long-term 5-aminosalicylic acid (5-ASA) is the first-line treatment for maintaining disease remission in UC.^2^^,^^3^

Globally, the incidence of UC is rising fastest in younger populations.^4^ Up to 30% of individuals with UC are diagnosed in childhood and young adulthood, and are more likely to have a severe disease course and years lived in disability relative to those diagnosed later in life.^4^ Therefore, disease control and maintaining remission is paramount for those diagnosed in early life. Despite this, estimates suggest medication adherence rates in young people are lower than adults with inflammatory bowel disease (IBD).^5^^,^^6^

International guidelines recommend 5-ASA treatment should start promptly after diagnosis and continue long term to maintain remission.^2^^,^^3^ Stopping 5-ASA maintenance treatment increases the risk of early disease relapse, flare frequency, and impaired quality of life.^7^ Studies also report long-term 5-ASA treatment may reduce the risk of colorectal cancer in individuals with UC.^8^ However, some patients will need escalated immunosuppressive medications. For this group, the benefits of concurrent 5-ASA treatment to maintain remission is less clear cut.^7^

Poor medication adherence also places a significant cost burden on society and healthcare services.^9^ In the UK, disease relapse has been shown to be associated with a 20-fold increase in costs for those who required admission to hospital when compared with a two-to-threefold increase for those who did not.^10^ Hence, medication adherence improves health outcomes and reduces resource use in health systems.

Being diagnosed with a lifelong condition such as UC can be challenging for adolescents, who are undergoing physiological, psychological, and social transitions to adulthood.^11^ It is important that young people learn to self-care in the early stages of long-term conditions. However, adolescents and young adults diagnosed with IBD have rated their knowledge about continuous medication as suboptimal.^12^ Adolescents and young adults diagnosed with IBD perceive adhering to daily medication as a burden,^13^ with previous studies reporting a wide variation of 2%–93% and lower adherence rates compared with adults.^1^^,^^14^^–^16 However, previous cross- sectional surveys, most commonly used to measure oral 5-ASA treatment adherence among adolescents, are subject to recall bias and may overestimate adherence.^1^ Far less is known about the extent and timing of discontinuation.

**Table:** How this fits in

Adolescents and young adults diagnosed with ulcerative colitis (UC) are recommended long- term maintenance treatment for disease control, but adherence rates in primary care are unknown. This observational cohort study using real-world data from primary care found one-quarter of newly diagnosed adolescents and young adults, aged 10–24 years, discontinued oral 5-aminosalicylic acid (5-ASA) maintenance treatment within 1 month of starting and two-thirds within 1 year. Young adults aged 18–24 years and those living in a deprived area were most likely to discontinue and have poor adherence to treatment. Having an acute flare-up of UC was linked to better adherence to oral 5-ASA maintenance treatment. The first year of starting lifelong therapies among individuals diagnosed with UC is a critical window to improve adherence for adolescents transitioning to young adulthood and those from deprived postcodes.

Qualitative studies of adolescents’ health literacy and decision making, such as adherence to medication, is influenced by social and structural determinants including health perception, health behaviours, and access to health services.^17^^,^^18^ However, this is not well described in young people with chronic conditions such as UC.

A population-based study was therefore designed using prospectively collected prescribing data to determine discontinuation and adherence to oral 5-ASA maintenance in the first year of treatment among adolescents and young adults aged 10–24 years diagnosed with UC. The secondary aim was to identify risk and protective factors associated with oral 5-ASA discontinuation and adherence.

## METHOD

The present study has been conducted as per recommendations provided by Strengthening the Reporting of Observational Studies in Epidemiology guidelines.^19^

### Data source

The Clinical Practice Research Datalink (CPRD) is one of the largest validated primary care research databases in the world.^20^ It contains the longitudinal, patient- level, anonymised electronic health records of 18 million patients from >700 general practices and is representative of the UK population.^20^ Primary care physicians use Read codes and prodcodes to record diagnoses and prescriptions, respectively. The database’s coding system has been validated for use in IBD and the reporting of medication adherence.^21^^,^^22^ English practice records are linked to the national hospital administrative database Hospital Episodes Statistics.

### Case definition and cohort construction

The study population included individuals diagnosed with UC who started oral 5-ASA maintenance treatment aged between 10 and 24 years within 6 months of their recorded UC diagnosis date. ‘Incident cases’ with a first-ever diagnosis code for UC at least 1 year after registering with an ‘up- to- standard’ practice between 1 January 1998 and 1 May 2016 were included, in accordance with previously validated and published methods.^21^ Patients were excluded if they had codes for both UC and Crohn’s disease, or indeterminate codes (for example, ‘non- specific colitis’ and ‘colitis NOS’). Patients who had a comorbid condition that might require regular or prolonged corticosteroid use, for example, chronic asthma, polymyalgia rheumatica, and organ transplants, were also excluded to avoid potential confounding, as steroid exposure in this group is not solely for IBD.^23^^,^^24^

Individuals were followed up from the oral 5-ASA start date for 1 year or until de-registration from their practice or death, whichever came first. Individuals whose coded diagnosis could not be determined and those who may have had oral 5-ASA treatment discontinued as a consequence of treatment escalation to immunomodulator therapy or if they had a colectomy during the study follow-up period were excluded. In addition, those with insufficient follow-up to identify whether they discontinued treatment during the first year (<90 days after the first break in treatment) were also excluded.^7^

### Outcomes

The primary outcome measure was time to oral 5-ASA discontinuation in the first year of maintenance treatment. Oral 5-ASA use was considered as continuous if prescriptions were within 90 days of each other. This cut-off was chosen as 75% of individuals were found to receive prescriptions every 2 months. This window allowed those who continue treatment but collect prescriptions late or use existing supplies to be included.

The secondary outcome was adherence to oral 5-ASA maintenance treatment, defined as the proportion of days covered (PDC) with oral 5-ASA medication. The PDC, a validated objective measure of medication adherence, was calculated by dividing the total number of days covered from prescribed oral 5-ASA by the duration of follow-up in the first year of treatment (days). The PDC was capped at 100% to ensure measurement of adherence was not overestimated.^25^

### Predictors of oral 5-ASA discontinuation and adherence

Potential predictors of oral 5-ASA discontinuation and adherence were identified from the biomedical literature and in consultation with a panel comprising specialist gastroenterologists, GPs, and researchers (the POP-IBD group).^26^^,^^27^

Sociodemographic predictors were:

- sex;
- age group when oral 5-ASA maintenance treatment started (10–14, 15–17, and 18–24 years); and
- Index of Multiple Deprivation (IMD) decile, which is a postcode-based measure of socioeconomic deprivation from 1 (least deprived) to 5 (most deprived).^26^

Health-related predictors included:

- having a psychiatric comorbidity, defined as any record of depression, anxiety, or antidepressant use during the study period; or
- poor health because of an acute flare of UC, defined as oral corticosteroid use within 3 months of UC diagnosis.

A proxy predictor of risky health behaviours was defined using proxy smoking status, defined as ‘smokers’, ‘ex-smokers’, or ‘non-smokers’ based on the most recent code for smoking status at UC diagnosis. Those with missing data on smoking have been shown to be either ‘never-smokers’ or ‘non-recent smokers’, and were therefore classed as ‘non- smokers’.^28^

Finally, the impact of era during which oral 5-ASA maintenance therapy was started was examined to account for secular changes in 5-ASA use over the study period (era 1: 1998–2002; era 2: 2003–2007; era 3: 2008–2012; and era 4: 2013–2016).

### Data analysis

Kaplan–Meier analysis was used to estimate time from the start of oral 5-ASA maintenance treatment to discontinuation. The PDC was calculated for each individual as a percentage of the first year covered with oral 5-ASA maintenance treatment.

In each model, where a potential predictor of oral 5-ASA discontinuation and adherence was examined, the probable blocks of mediators were determined and potential confounders adjusted for. Cox regression analysis was used to determine hazard ratios (HRs) for the risk of oral 5-ASA discontinuation in the year after starting maintenance treatment. Cox regression analysis was used so it was possible to account for the time each individual was at risk of oral 5-ASA discontinuation. Simple and multiple linear regression analysis was used to determine predictors associated with adherence to oral 5-ASA in the first year of maintenance treatment.

All analyses were performed using Stata (version 17) software.

## RESULTS

**Figure 1.**
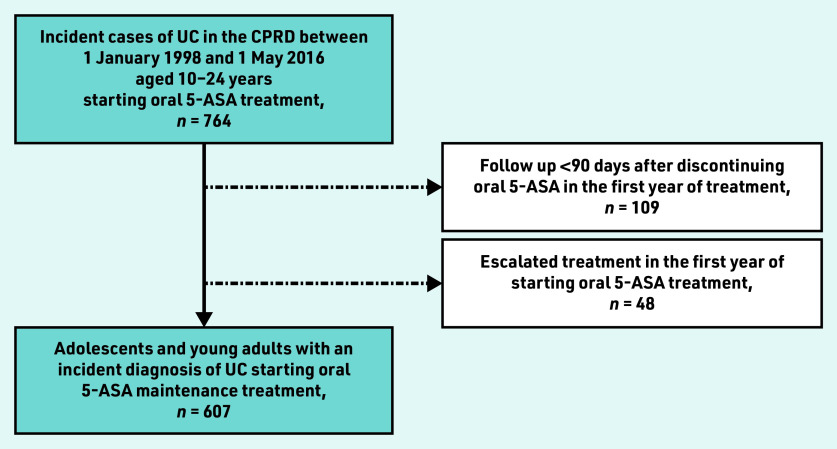
*Cohort construction.* *5-ASA = 5-aminosalicylic acid. CPRD = Clinical Practice Research Datalink. UC = ulcerative colitis.*

A total of 607 adolescents and young adults were identified with an incident diagnosis of UC who started oral 5-ASA maintenance treatment, excluding 48 individuals who required treatment escalation in the first year of starting oral 5-ASA and 109 ineligible individuals because of insufficient follow-up (Figure 1). Baseline characteristics of the study population can be found in Table 1.

**Table 1.** Baseline characteristics of study population

**Characteristic**	***n* (%)**
**Sex**	
Male	327 (56)
Female	280 (46)

**Age group at oral 5-ASA start date, years**	
10 to 14	86 (14)
15 to 17	99 (16)
18 to 24	422 (70)

**Era of UC diagnosis**	
Era 1: 1998–2002	56 (9)
Era 2: 2003–2007	134 (22)
Era 3: 2008–2012	169 (28)
Era 4: 2013–2016	248 (41)

*5-ASA = 5-aminosalicylic acid. UC = ulcerative colitis.*

**Figure 2.**
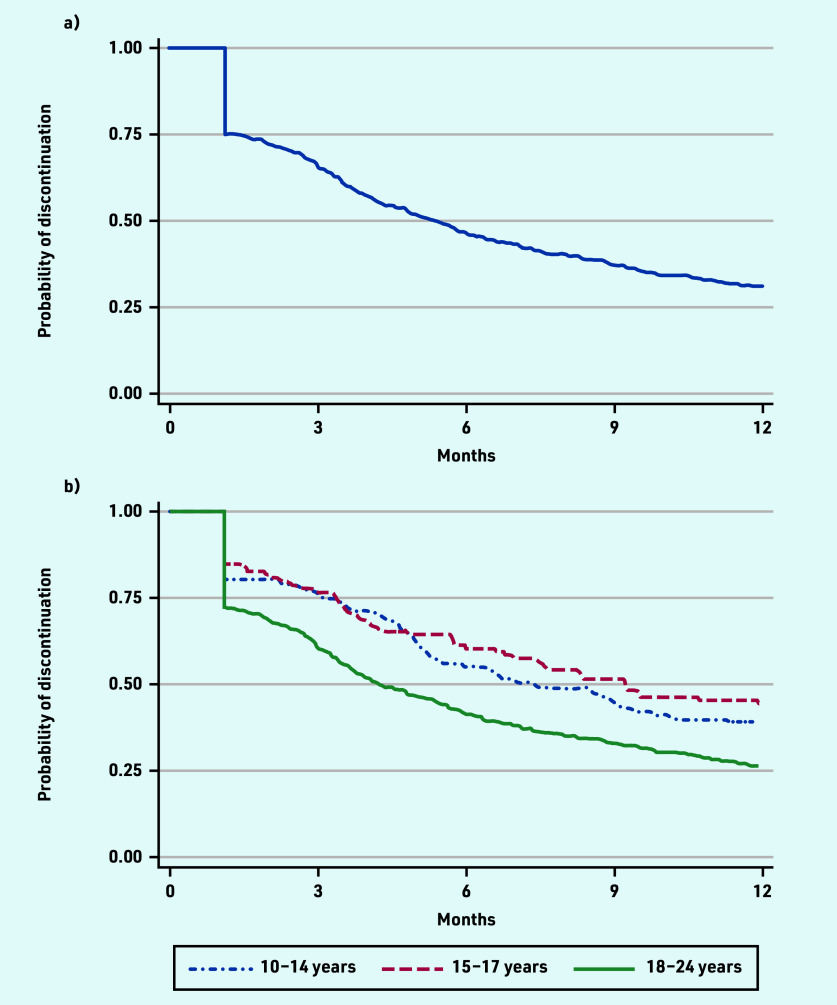
*Kaplan–Meier curve demonstrating probability of oral 5-ASA discontinuation in the first year of treatment for a) all adolescents and young adults, and b) by age at oral 5-ASA initiation. 5-ASA = 5-aminosalicylic acid.*

Overall, 69% (*n* = 419) of individuals discontinued oral 5-ASA maintenance treatment within 1 year of starting (Figure 2a). One-quarter (*n* = 152) of the cohort discontinued treatment by day 34. The median time to discontinuation was 162 days (data not shown). Among individuals who discontinued oral 5-ASA, 90% (*n* = 379/420) had no subsequent prescription in the first year of treatment.

Discontinuation rates at 1 year were lowest in younger adolescents (56% in those aged 15–17 years and 61% in those aged 10–14 years) and highest among young adults aged 18–24 years (74%) (Figure 2b). Among young adults aged 18–24 years, 28% discontinued oral 5-ASA maintenance treatment after a single prescription, compared with 19% and 15% of adolescents starting treatment at 10–14 and 15–17 years, respectively (data not shown).

Mean adherence for the study population in the first year of oral 5-ASA maintenance treatment was 72% (95% confidence interval [CI] = 70 to 75), equivalent to just under 9 months’ duration in the first 12 months of treatment. Adherence fell with older age at oral 5-ASA initiation. This was 80% (95% CI = 74 to 86) among adolescents aged 10–14 years, 78% (95% CI = 72 to 84) among adolescents aged 15–17 years, and 69% (95% CI = 66 to 72) among young adults aged 18–24 years (data not shown).

### Risk and protective factors for oral 5-ASA discontinuation and adherence

Young adults aged 18–24 years starting oral 5-ASA were more likely to discontinue maintenance treatment in the first 12 months compared with adolescents aged 10–14 years (adjusted hazard ratio [aHR] 1.43, 95% CI =1.04 to 1.97). Individuals living in deprived versus affluent postcodes were more likely to discontinue oral 5-ASA maintenance treatment (aHR 1.46, 95% CI = 1.10 to 1.92). Individuals who experienced a UC flare requiring early oral corticosteroids were less likely to discontinue oral 5-ASA maintenance treatment compared with individuals who did not require corticosteroids (aHR 0.68, 95% CI = 0.51 to 0.90; Table 2).

**Table 2.** Predictors of oral 5-ASA treatment discontinuation^a^

**Predictor**	**Unadjusted model,** **HR (95% CI)**	**Fully adjusted** **model, HR (95% CI)**
**Age group, years**		
10–14	Reference	Reference
15–17	0.88 (0.62 to 1.28)	0.84 (0.56 to 1.26)
18–24	**1.42 (1.06 to 1.91)**	**1.43 (1.04 to 1.97)**

**Sex**		
Male	Reference	Reference
Female	0.95 (0.79 to 1.16)	0.98 (0.80 to 1.21)

**IMD^b^**		
IMD 1–3	Reference	Reference
IMD 4 and 5	**1.37 (1.06 to 1.77)**	**1.46 (1.10 to 1.92)**

**Smoking status**		
Never	Reference	Reference
Ex-smoker	0.94 (0.64 to 1.38)	0.86 (0.55 to 1.34)
Current	1.37 (0.92 to 2.06)	1.22 (0.79 to 1.88)

**Psychiatric comorbidity of depression, anxiety, or antidepressant use^c^**		
No	Reference	Reference
Yes	1.16 (0.91 to 1.48)	1.02 (0.77 to 1.37)

**Era of diagnosis**		
Era 1: 1998–2002	Reference	Reference
Era 2: 2003–2007	1.04 (0.70 to 1.56)	1.03 (0.69 to 1.54)
Era 3: 2008–2012	1.13 (0.77 to 1.67)	1.14 (0.78 to 1.69)
Era 4: 2013–2016	**1.47 (1.00 to 2.14)**	1.46 (0.99 to 2.14)

**UC flare with early corticosteroid use^d^**		
No	Reference	Reference
Yes	**0.65 (0.50 to 0.85)**	**0.68 (0.51 to 0.90)**

a *The multiple regression model adjusted for all listed variables in table.*

b *IMD categories 4 and 5 (most deprived) versus IMD categories 1, 2, and 3 (least deprived). Data available for 60% of patients.*

c *Individuals with a code for depression, depressive symptoms, anxiety, symptoms of anxiety, or antidepressant medication use (excluding low- dose tricyclic antidepressants) at any point during or before the year following oral 5-ASA start date.*

d *Corticosteroid use within the 3 months of diagnosis of UC — a proxy marker of disease severity. 5-ASA = 5-aminosalicylic acid. Bold = statistically significant. HR = hazard ratio. IMD = Index of Multiple Deprivation. UC = ulcerative colitis.*

Predictors of lower adherence included starting treatment during young adulthood (aged 18–24 years) and living in poorer postcodes. Individuals who had an acute flare and required early corticosteroid use had higher adherence to oral 5-ASA maintenance treatment (Table 3).

**Table 3.** Predictors of adherence to oral 5-ASA treatment^a^

**Predictor**	**Unadjusted, coefficient (95% CI)**	**Fully adjusted, coefficient (95% CI)**
**Age group, years**		
10–14	Reference	Reference
15–17	−0.02 (−0.11 to 0.07)	−0.01 (−0.10 to 0.09)
18–24	**–0.11 (−0.18 to −0.04)**	**–0.12 (−0.20 to −0.04)**

**Sex**		
Male	Reference	Reference
Female	0.002 (−0.05 to 0.05)	−0.01 (−0.06 to 0.04)

**IMD^b^**		
IMD 1–3	Reference	Reference
IMD 4 and 5	**–0.10 (−0.17 to −0.03)**	**–0.11 (−0.18 to −0.03)**

**Smoking status**		
Never	Reference	Reference
Ex-smoker	−0.003 (−0.10 to −0.01)	0.04 (−0.08 to 0.15)
Current	−0.10 (−0.21 to −0.02)	−0.04 (−0.16 to 0.08)

**Psychiatric comorbidity of depression, anxiety, or antidepressant use^c^**		
No	Reference	Reference
Yes	−0.04 (−0.11 to 0.02)	−0.02 (−0.10 to 0.05)

**Era of diagnosis**		
Era 1: 1998–2002	Reference	Reference
Era 2: 2003–2007	0.01 (−0.09 to 0.11)	0.02 (−0.08 to 0.12)
Era 3: 2008–2012	−0.01 (−0.11 to 0.08)	−0.01 (−0.11 to 0.08)
Era 4: 2013–2016	−0.02 (−0.11 to 0.08)	−0.01 (−0.10 to 0.08)

**UC flare with early corticosteroid use^d^**		
No	Reference	Reference
Yes	**0.12 (0.05 to 0.18)**	**0.11 (0.04 to 0.17)**

a *The multiple regression model adjusted for all listed variables in table.*

b *IMD categories 4 and 5 (most deprived) versus IMD categories 1, 2, and 3 (least deprived). Data available for 60% of patients.*

c *Individuals with a code for depression, depressive symptoms, anxiety, symptoms of anxiety, or antidepressant medication use (excluding low- dose tricyclic antidepressants) at any point during or before the year following oral 5-ASA start date.*

d *Corticosteroid use within the 3 months of diagnosis of UC — a proxy marker of disease severity. 5-ASA = 5-aminosalicylic acid. Bold = statistically significant. IMD = Index of Multiple Deprivation. UC = ulcerative colitis.*

Psychiatric comorbidity of depression, anxiety, or antidepressant use was not associated with discontinuation or adherence to oral 5-ASA maintenance treatment (Tables 2 and 3).

## DISCUSSION

### Summary

One-quarter of adolescents and young adults newly diagnosed with UC discontinued oral 5-ASA maintenance treatment within 1 month and two- thirds within 1 year. Of those who discontinued, 90% had no subsequent oral 5-ASA prescription in the first year of treatment. Adolescents and young adults adhered to oral 5-ASA maintenance treatment for an average of 9 months in the first year of starting.

Young adults aged 18–24 years and those living in deprived postcode areas were 43% and 46% more likely to discontinue oral 5-ASA maintenance treatment, respectively. By contrast, those with an acute flare who required corticosteroids during the early stages of diagnosis were more likely to continue and adhere to oral 5-ASA maintenance in the first year of treatment.

### Strengths and limitations

This is the first population-based study, to the authors’ knowledge, to objectively examine discontinuation and adherence of oral 5-ASA maintenance treatment among adolescents, including the transition period into early adulthood. Real-world data were used, drawn from a large nationally representative validated primary care database that captures a high level of community prescribing. CPRD data are collected at the time of consultation or prescription and are therefore independent of referral centre, recall, or participant selection bias.

Important limitations in the current study are an assumption that repeat prescriptions issued in primary care were indicative of adherence. Hence, discontinuation rates may be higher and adherence lower than reported in this study. In the UK, hospital outpatient prescribing is highly regulated and primary care physicians are responsible for prescribing for patients with chronic conditions in the community. However, it was not possible to capture a small minority of short-term prescriptions given in hospital at discharge after admission, in the private sector, or obtained overseas.

### Comparison with existing literature

To the authors’ knowledge, the current study is among the largest population- based cohort studies of incident cases of adolescents and young adults diagnosed with UC. Previous studies of oral 5-ASA adherence among adolescents have reported lower objective adherence rates of 52%–71%.^14^^,^^29^ These studies have focused on populations drawn from single centres or have been about individuals aged <18 years, and do not capture this transition period to young adulthood.^1^^,^^14^

In the current study, strict inclusion criteria were applied for the cohort construction to ensure robust case ascertainment. The sample size of 607 new cases is consistent with the incidence of UC of approximately 10–20 patients per 100 000 per year reported elsewhere.^30^

The current finding that approximately one-quarter of patients discontinue oral 5-ASA maintenance treatment after 1 month is consistent with discontinuation rates in adults with IBD.^31^ This study’s objective estimate of 72% oral 5-ASA adherence among adolescents and young adults is much lower than self-reported estimates of 93%–96% from previous studies that are subject to recall bias.^32^^,^^33^ Using electronic records for measuring discontinuation and adherence overcomes reporting bias seen in previous studies because patients and families are not aware they are being evaluated.^1^

The relapsing and remitting course of UC may influence the perceived importance of maintenance treatment. The estimated mean time to control symptoms with 5-ASA in UC is reported to be 4 weeks.^34^ It may be that, once symptoms resolve, the motivation to persist with treatment falters. Some individuals may not perceive themselves to be at risk of long-term consequences of the illness, an important motivator to continue treatment. Conversely, those who experience disease flares may find it easier to accept the need for maintenance treatment. This may explain this study’s findings that the risk of oral 5-ASA treatment discontinuation was lower and adherence higher among those who had a flare treated with corticosteroids during the early stages of disease.

The findings from the current research of higher risk of treatment discontinuation and poor adherence during the transition from adolescence to adulthood may be explained by the loss of support from caregivers who encourage adherence in adolescents and provide financial and practical support.^35^ Previous literature is conflicting about the impact of poor mental health on medication adherence.^32^^,^^36^^,^^37^ In the current study it was not found that adolescents and young adults with depression, anxiety, or antidepressant use had poor adherence or discontinued oral 5-ASA maintenance treatment. It is possible that the association between poor mental health was underestimated, as depression and anxiety may not be detected or coded for in primary care, and low medication adherence is associated with concurrent psychiatric comorbidity.^37^

### Implications for research and practice

The current findings may mean that adolescents and young adults diagnosed with UC are at a high risk of early relapse*.* Non-adherence to 5-ASA (PDC <80%) is associated with a five-fold risk of disease relapse compared with those whose adherence is over 80%.^38^ The current study found a mean adherence rate of 72% among adolescents and young adults in their first year of oral 5-ASA maintenance treatment. If clinicians are unaware of suboptimal adherence to first-line medication they may incorrectly assume therapy has failed, which may lead to unnecessary escalation in treatment and avoidable steroid use that remains high in UC.^39^ The methods used in the current study have application for future studies and clinical audits to assess adherence to long-term medication using electronic records. Future longitudinal studies using linked data on prescribed treatments in primary care and hospital settings are needed to identify the impact of poor adherence and discontinuation on clinical outcomes of UC.

Clear communication about the importance of regular maintenance for adolescents and young adults should be coupled with an action plan for managing their chronic condition. For example, it is a common misconception that treatment should be discontinued when symptoms resolve in other inflammatory conditions such as asthma. Structured transition programmes from paediatric to adult health care can empower adolescents by providing them with the knowledge and skills to manage their own disease.^40^^,^^41^ Although developing patient autonomy for managing their condition is a prerequisite for good adherence, the current study supports findings of a recent patient survey and self- assessment of 134 IBD services in the UK that found two-thirds of services scored below standard on their transition care pathways.^42^

A survey of GPs in the 2017 Royal College of General Practitioners IBD ‘Spotlight’ project reported 50% lacked confidence in managing IBD.^43^ Prescribing physicians are often under pressure to reduce health system costs by issuing shorter prescriptions or deprescribing long-term medications. However, it has been argued that this may have adverse long-term health and economic consequences for patients with chronic conditions.^43^ Based on the findings in the current study, the authors recommend better integration to agree roles and responsibilities between primary and secondary care as this could improve adherence during the early stages of starting treatment.

This study highlights the need to address socioeconomic disparities that could be driving oral 5-ASA discontinuation and low adherence among adolescents and young adults who may struggle with meeting the costs of long-term prescriptions.^44^ In the US, an increase in each dollar of medication co-payment decreases medication adherence rates by 0.4%.^44^ Even in the UK, where 98% of patients have access to universal healthcare coverage, one-third of individuals with IBD paying prescription charges do not pick up a prescription because of cost, and 15% take medicines less frequently than required to reduce costs.^45^

In conclusion, adolescents and young adults diagnosed with UC starting oral 5-ASA maintenance treatment are at risk of discontinuation and poor adherence, a majority of whom discontinue early in the first year of treatment. These findings illustrate the importance of clinicians ensuring careful follow-up within the first year when prescribing lifelong therapies for adolescents and young adults who are diagnosed with UC, particularly adolescents transitioning to young adulthood and those living in deprived areas.

## Data

Data may be obtained from the Clinical Practice Research Datalink and are not publicly available.
